# Effect of short implant crown-to-implant ratio on stress distribution in anisotropic bone with different osseointegration rates

**DOI:** 10.1186/s12903-023-03379-z

**Published:** 2023-09-20

**Authors:** Xi Yuan, Yuchen Liu, Yunhe Yang, Mingfa Ren, Lailong Luo, Lang Zheng, Yang Liu

**Affiliations:** 1 Affiliated Hospital of Shaanxi University of Chinese Medicine, Xianyang, 712000 China; 2 Dalian University of Technology, Dalian, 116000 China; 3 Dalian Stomatological Hospital, Dalian, 116000 China; 4 Dalian University, Dalian, 116000 China; 5 Department of Prosthodontics, Dalian Stomatological Hospital, 935 Changjiang Road, Shahekou District, Dalian, 116000 China

**Keywords:** Short implant, Osseointegration rate, Crown-to-implant ratio, Three-dimensional finite element

## Abstract

**Objective:**

This study aimed to provide evidence for the clinical application of single short implants by establishing an anisotropic, three-dimensional (3D) finite element mandible model and simulating the effect of crown-to-implant ratio (CIR) on biomechanics around short implants with different osseointegration rates.

**Methods:**

Assuming that the bone is transversely isotropic by finite element method, we created four distinct models of implants for the mandibular first molar. Subsequently, axial and oblique forces were applied to the occlusal surface of these models. Ultimately, the Abaqus 2020 software was employed to compute various mechanical parameters, including the maximum von Mises stress, tensile stress, compressive stress, shear stress, displacement, and strains in the peri-implant bone tissue.

**Results:**

Upon establishing consistent osseointegration rates, the distribution of stress exhibited similarities across models with varying CIRs when subjected to vertical loads. However, when exposed to inclined loads, the maximum von Mises stress within the cortical bone escalated as the CIR heightened. Among both loading scenarios, notable escalation in the maximum von Mises stress occurred in the model featuring a CIR of 2.5 and an osseointegration rate of 25%. Conversely, other models displayed comparable strength. Notably, stress and strain values uniformly increased with augmented osseointegration across all models. Furthermore, an increase in osseointegration rate correlated with reduced maximum displacement for both cortical bone and implants.

**Conclusions:**

After fixing osseointegration rates, the stress around shorter implants increased as the CIR increased under inclined loads. Thus, the effect of lateral forces should be considered when selecting shorter implants. Moreover, an implant failure risk was present in cases with a CIR ≥ 2.5 and low osseointegration rates. Additionally, the higher the osseointegration rate, the more readily the implant can achieve robust stability.

## Introduction

Although implant technology has gained more momentum in recent years, the direct placement of conventional implants is difficult due to the complex adjacent anatomical structures like the maxillary sinus and mandibular nerve, as well as inadequate working conditions such as insufficient alveolar bone height. These limitations can be overcome by various procedures like maxillary sinus augmentation, inferior alveolar neural tube transposition, and bone augmentation. However, these techniques have several disadvantages, such as the increased risk of trauma and postoperative complications, high costs, and long treatment duration. Oral implants have made numerous advances in recent years. In particular, they aim to optimize implant design, implant surface, and connections between the implant and abutment. The implant has not only achieved improved biomechanics but has also increased the contact area with the bone implant [1]. Therefore, the usage of short implants has become a viable option in such cases. The 2018 International Team for Implantology (ITI) Consensus Conference defined short implants as implants ≤ 6 mm in length [2].

Several studies reporting newer implant surface treatment and short implant design revealed that short implants had similar success rates compared with conventional implants and exhibited good clinical effectiveness [3–5]. However, a comparison of short implants (≤ 6 mm) with conventional implants, followed up for 1–5 years, revealed a higher risk of failure for short implants [6, 7]. Therefore, clinicians should consider all potential risk factors associated with the usage of short implants^5^. Among them, the crown-to-implant ratio (CIR) is a clinical design factor that affects the effectiveness of single short implants [8].

The cross-to-implant ratio, also known as the crown-to-implant ratio (CIR), pertains to the proportion between the height of the crown and the length of the implant. Short implants find predominant use in posterior areas characterized by inadequate vertical alveolar bone height. Their utilization results in an elevated CIR. The connection between crown length and implant length, under the influence of occlusal forces, can be likened to that of a Class I lever. Consequently, increased CIR might lead to occlusal overload, and the implant adjacent to the vertical cantilever extension displays loss of osseointegration [9]. A significant crown-to-implant ratio will not solely contribute to diminished implant stability but will also lead to marginal bone resorption [10]. Certain researchers posit that the prospective survival rate of short implants, particularly those of a length equal to or less than 10 mm, is limited. This limitation is attributed to their substantial crown-root ratio, resulting in reduced clinical predictability [11]. However, Garaicoa-Pazmiño et al. [12], in a meta-analysis for evaluating the marginal bone resorption of short implants, found that the greater the CIR, the lesser the bone resorption. Several studies have indicated that the force exerted by biting primarily focuses on the cortical bone and exhibits a weak correlation with implant length [13]. Furthermore, no statistical correlation has been observed between marginal bone loss and the implant’s CIR [14]. Furthermore, a meta-analysis conducted by Henny J et al. in 2018 revealed that there existed no noteworthy disparity in the frequency of mechanical and biological complications when the crown-to-root ratio fell within the range of 0.9 to 2.2 [15]. Therefore, the underlying effects of CIR on marginal bone resorption of short implants require further investigation.

The periodontal ligaments act as shock absorbers and contain several mechanoreceptors that interact with occlusal forces accordingly. However, in the absence of periodontal ligaments around implants, occlusal forces will be transmitted directly to the surrounding bone. Thus, the implants exhibit low tactile sensitivity and proprioceptive feedback, which often leads to increased biological and mechanical complications. Additionally, long-term implant survival also depends on the implant’s stability in alveolar bones. As this stability is achieved by osseointegration, an implant’s success is related to biological and biomechanical factors as well as clinical settings. Hence, the biomechanics of peri-implant bones is extremely critical for evaluating the reliability and efficacy of the implant [16].

The finite element method (FEM) is an effective technique to analyze biomechanical properties and has been widely used in oral implant designs [17]. Finite element analysis (FEA) is the process of using models to simulate mechanical problems in a specific scenario. Thus, it allows us to perform multiple tests over a short period and arbitrarily modify key factors in a manner that cannot be achieved by animal or clinical studies. FEA has the capacity to simulate intricate models, encompassing their diverse mechanical attributes, thus facilitating comprehensive analysis and potential refinement as needed [18]. Nevertheless, the precision of FEA outcomes is significantly contingent on the computational parameters. As the finite element method functions as a numerical computational technique, it transforms the actual structure into a discrete model comprised of numerous small units. Subsequently, it solves physical parameters like stress and strain for each unit via mathematical equations. Given its approximative nature, it becomes imperative to consider the value that yields minimal error across the entire solution [19]. The accuracy of this approach is primarily reliant on the precision of the model, material and tissue characteristics, boundary and load conditions, cell type, grid sensitivity, and contact definition [20].

Previous FEM studies have analyzed the properties of bones as osseointegrated and isotropic. A quantitative clinical assessment showed an absence of complete osseointegration and suggested that bones exhibited different mechanical properties when measured in different directions [21]. Thus, these findings confirmed that the peri-implant osseointegration rate cannot reach 100%, and the bone is an anisotropic tissue. Different studies have utilized varied osseointegration rates, such as randomly connecting the bone-implant interface according to the designated osseointegration rate and establishing uniform pore-like structural bone tissue [22, 23]. Although these methods utilized the osseointegration rates, they were unrealistic and did not consider the anisotropy characteristics of bones. Additionally, few studies only considered the anisotropic characteristics and bone-deficient osseointegration rates [24, 25]. Therefore, the results obtained by the previous simplified method were not accurate, thus, limiting its clinical application.

In our study, a transition region of the bones was defined to represent partial osseointegration that stimulates the condition of peri-implant bones accurately. As the cortical bone’s elastic modulus was similar to that of the cancellous bone in buccolingual and mesiodistal directions, we assumed that the bones were transversely isotropic and longitudinally anisotropic [16]. Additionally, implant restorations in atrophic jaws are challenging because the atrophic jaws contain Class III bones with low bone mass and poor quality [26]. We also used CIR as an independent factor to simulate stresses and strains in peri-implant bones caused by occlusal loading in Class III bones and to analyze the effects of osseointegration degree.

However, there is a lack of biomechanical studies on the CIR of short implants under different osseointegration rates in previous studies. Hence, the objective of this experiment was to assess the impact of the crown-to-implant ratio on bone stress distribution around short implants, considering varying bone binding rates. This endeavor aims to provide insights for designing short implants for diverse bone healing circumstances.

## Materials and methods

### Experimental equipment

We used the following hardware: Dell commercial desktop computer, 64 GB RAM, 128 GB hard drive, and Win7 64-bit operating system, as well as a 3Shape R700 scanner (3Shape, Copenhagen, Denmark).

We used the following software: SolidWorks 2019 3D computer-aided design software (SolidWorks Corp, USA), Geomagic studio2012 software (Geomagic, Inc), HyperMesh 12.0 (Altair, Inc), and Abaqus 2020 (Dassault, USA).

### Grouping

We investigated the stress distribution around short implants in four different CIRs under Class III bone conditions with varying osseointegration rates. The short implants used were: Straumann columnar soft tissue horizontal implants with a size of 4.8 × 6 mm. Furthermore, the implants were divided into four model groups with CIRs of 1, 1.5, 2, and 2.5, respectively.

### Establishment of models

#### Establishment of the local mandible model

**Fig. 1:**
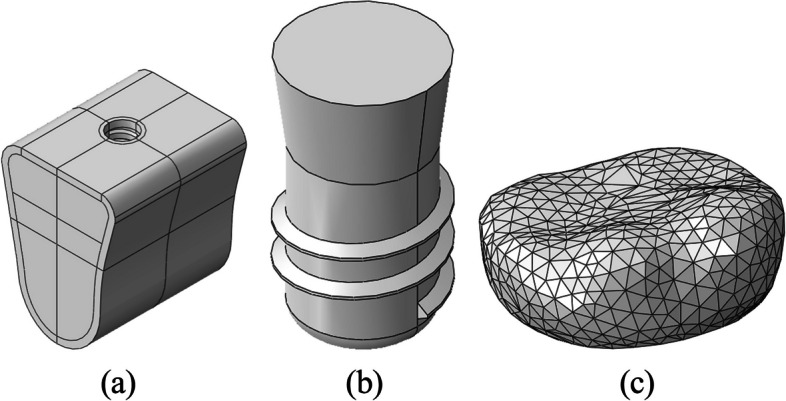
**a** Local mandible model, **b** An implant model, **c** A crown model

Solidworks 2019 three-dimensional (3D) software was used for establishing a dentition defect model in the posterior mandibular region. The model’s measurements, i.e., the jaw height, mesiodistal diameter, and buccolingual diameter at the alveolar crest, were established at 23.1 mm, 20 mm, and 17 mm, respectively (Fig. 1).

#### Establishment of the implant model

We simulated the biomechanical behavior of Straumann columnar soft tissue horizontal implants (Straumann Company, Switzerland). A short cylindrical implant model was created, as per the manufacturer’s data (diameter and length of 4.1 mm and 6 mm, thread spacing and depth of 1.25 mm and 0.38 mm, smooth neck diameter of 4.8 mm, and height of 2.8 mm). Using the Solidworks2019 software’s drawing function, a two-dimensional cross-sectional pattern and a 3D implant model without a threaded structure were obtained after 360° of rotation around the central axis. Furthermore, the thread tool in Solidworks2019 software was used for drawing the thread with corresponding parameters (Fig. 1).

#### Establishment of the crown model

The resin crown of the mandibular first molar was scanned by the 3Shape R700 laser scanner to generate a digitized model that was stored in the STL format. Moreover, the mandibular first molar model files were imported into HyperMesh 12.0, and models with four different crown heights were obtained by stretching according to the 4 CIRs in the experimental design. Subsequently, four models with different crown heights were imported into the Geomagic studio2012 software for simplification, i.e., they were optimized, smoothed, and exported in the STEP format (Fig. 1).

### 3D FEA

#### Model assembly

**Fig. 2:**
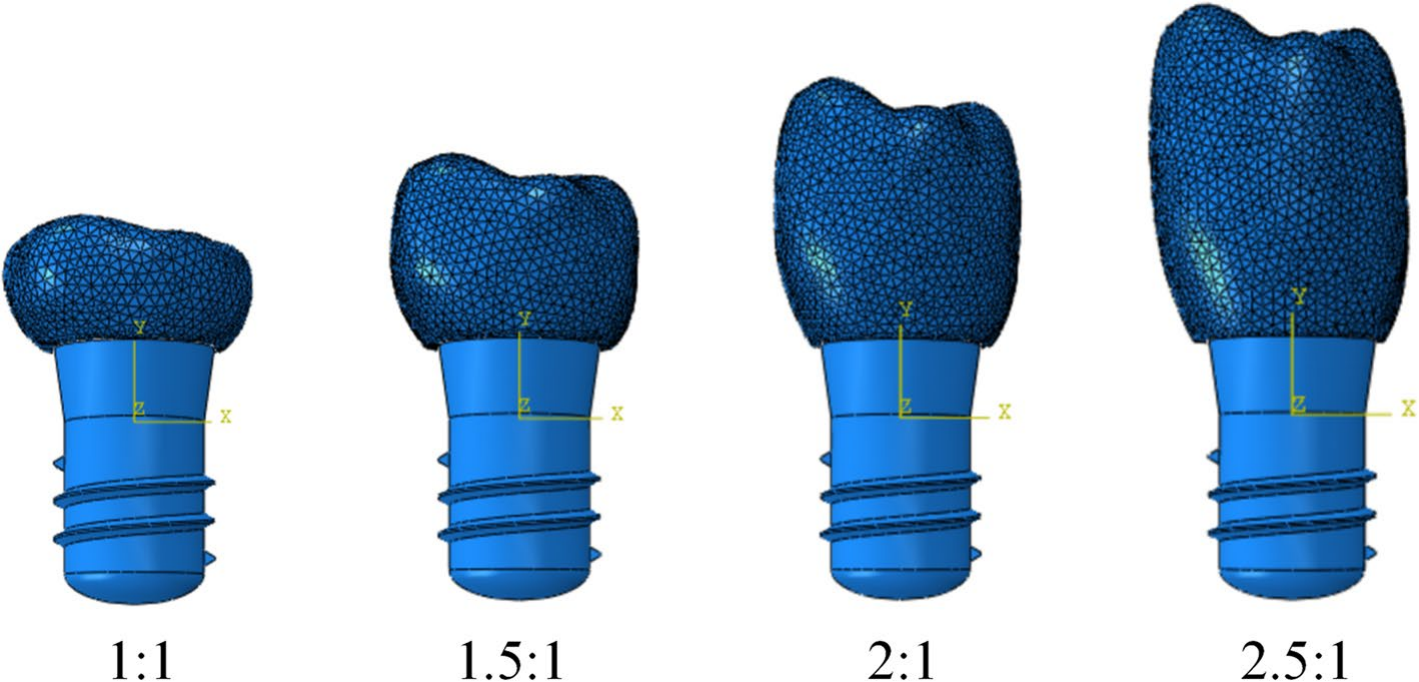
Four models with different CIRs

**Fig. 3:**
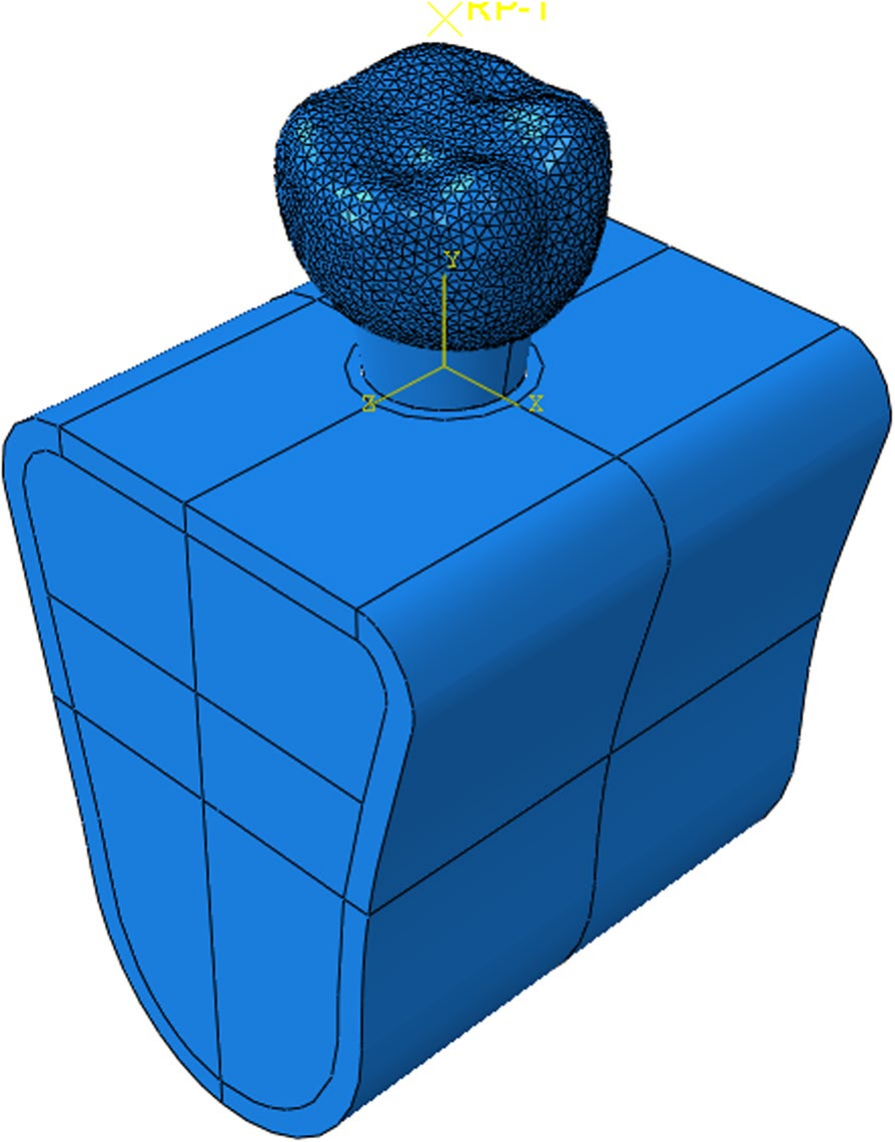
Completion of model assembly

The implant model, local mandible model, and four models with different crown heights were imported into Abaqus 2020 software and assembled according to the study groups in the assembly module (Fig. 2). Moreover, the models were adjusted to the required specifications (such as implant position, long axis direction, etc.) using geometric trimming tools, as shown in Fig. 3.

#### Assignment of material properties

**Fig. 4:**
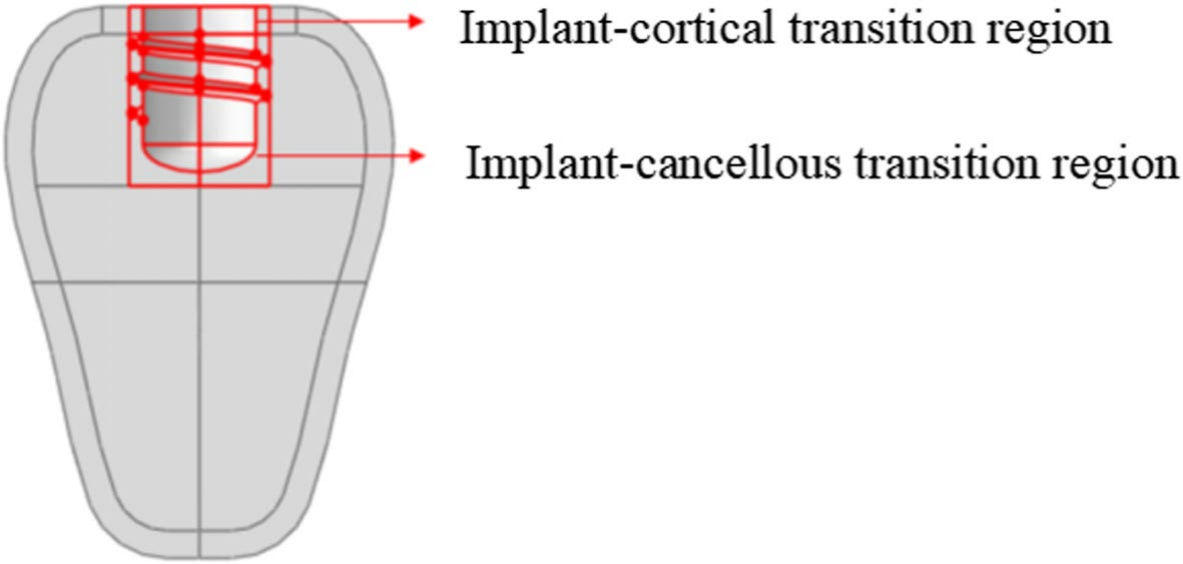
Division of local mandibular regions

In the Abaqus 2020 computational software, the bony regions were divided into cortical, cancellous, and osseointegrated regions [16, 27], as seen in Fig. 4. The Class III bone was characterized by the presence of cortical bone having a thickness of 1 mm, a dense cancellous bone, and an osseointegrated region 0.5 mm from the threaded implant floor.

The material properties were further assigned to the implant and crown according to the elastic modulus and Poisson’s ratio data provided in the literature. The implant material was titanium alloy, E = 110, GPa, v = 0.35, while the crown material was zirconia all-ceramic with E = 140,000 and GPa, v = 0.35. However, the material parameters in the osseointegrated region were reduced according to the osseointegration rates; the material properties of the mandible are shown in Table 1 [16].

**Table 1:** Experimental materials’ parameters

Properties	Cancellous bone				Cortical bone			
	25%	50%	75%	100%	25%	50%	75%	100%
*E*_*x*_*(MPa)*	287	574	861	1148	3150	6300	9450	12600
*E*_*y*_*(MPa)*	52.5	105	157.5	210	3150	6300	9450	12600
*E*_*z*_*(MPa)*	287	574	861	1148	4850	9700	145500	19400
*v*_*xy*_	0.05	0.05	0.05	0.05	0.05	0.3	0.3	0.3
*v*_*xz*_	0.32	0.32	0.32	0.32	0.253	0.253	0.253	0.253
*v*_*yz*_	0.01	0.01	0.01	0.01	0.253	0.253	0.253	0.253
*G*_*xy*_*(MPa)*	17	34	51	68	1212.5	2425	3637.5	4850
*G*_*xz*_*(MPa)*	108.5	217	325.5	434	1425	2850	4275	5700
*G*_*yz*_*(MPa)*	17	34	51	68	1425	2850	4275	5700

#### Setting boundary conditions and interface relationship

**Fig. 5:**
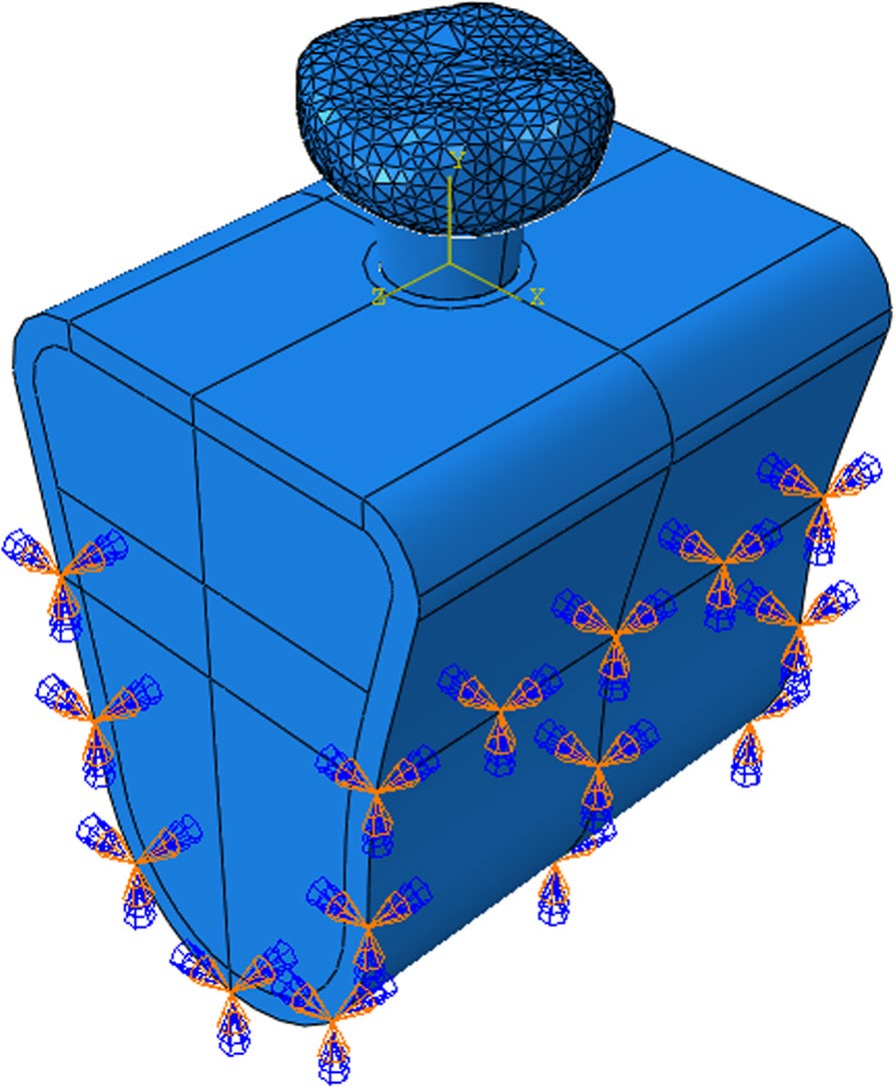
A schematic of boundary conditions

As seen in Fig. 5, full fixation constraint was applied to the outer surface of the lower jaw, i.e., no displacement happened after applying loads. After the application of tie constraint to the implant-mandible interface, the implant could not slide relative to the mandible [16, 28, 29].

#### Applying loads

First, the crown’s occlusal surface was coupled to a point, and the following loads were applied to the central fossa according to the mean masticatory force mentioned in a study [30]: vertical load: 150 N, parallel to the long axis of the implant, and inclined load: 50 N, 45° to the long axis of the implant.

#### Meshing

We used tetrahedral elements for meshing. In order to calculate the accuracy of our results, the mesh size of the cortical and osseointegrated transition zone was set as 0.2 mm for, 0.4 mm for cancellous bone, and 0.5 mm for the implant. The number of generated elements and nodes is shown in the Table 2. Finally, a mesh convergence test was performed to check whether the mesh met the calculation requirements.

**Table 2:** Number of mesh elements and nodes of models

Groups	1	1.5	2	2.5
Elements	251694	265128	273512	283953
Nodes	352908	372470	383788	398455

#### 3D FEA calculation

The calculation was performed for each model, and stress cloud maps were plotted in the Abaqus 2020 software.

### Analysis of physicomechanical properties

The maximum von Mises stress, tensile stress, compressive stress, shear stress, maximum displacement, maximum strain, and stress distribution map calculated for each group were analyzed. Although the yield strength of cortical bone was 160 MPa, the yield strength of different osseointegration rates changed proportionally with the magnitude of the osseointegration rate [16]. The ultimate tensile and compressive strengths of the cortical bone were approximately 100–121 MPa and 167–173 MPa, respectively [31].

## Results

### Maximum von Mises stress and stress distribution

**Fig. 6:**
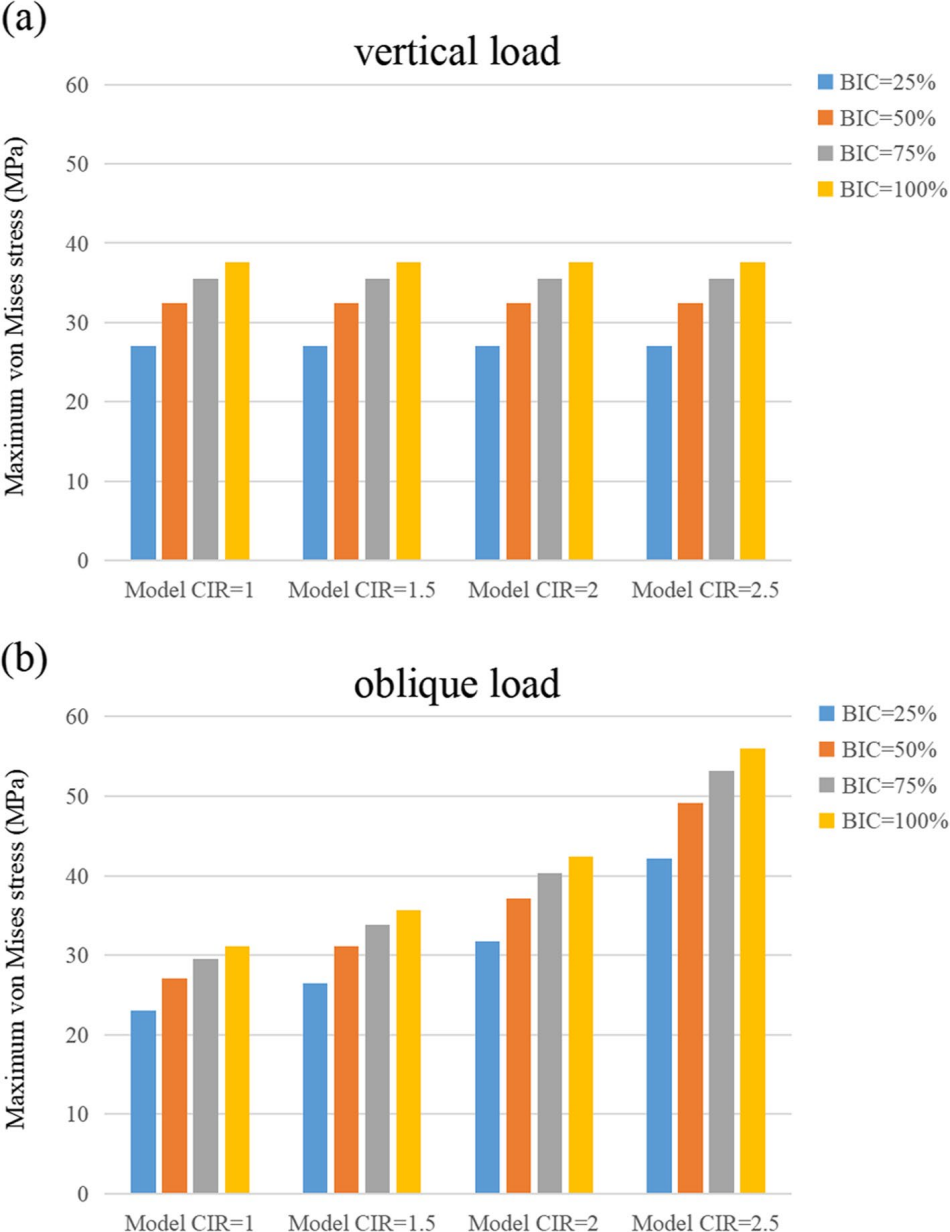
Maximum von Mises stress on the cortical bone

Figure 6 shows the changes in maximum von Mises stress for all the models under vertical and inclined loads, respectively. In both loading conditions, the models’ cortical bone was subjected to the maximum von Mises stress within its yield strength range, except for the model with a CIR of 2.5 and an osseointegration rate of 25%, as shown in Table 3. After fixing the osseointegration rates, similar stress values were obtained from the models with different CIRs under vertical loads. Moreover, after fixing the osseointegration rate, the maximum von Mises stress on the cortical bone under inclined loads increased as the CIR escalated. Additionally, an increase in osseointegration rate also enhanced the maximum von Mises stress by 30% within the same CIR.

**Table 3:** Maximum von Mises stress on the cortical bone

		Maximum von Mises stress (MGPa)	
		Vertical load	Inclined load
BIC = 25%	1:1 C/I	27.06	23.00
	1.5:1 C/I	27.06	26.48
	2:1 C/I	27.05	31.71
	2.5:1 C/I	27.05	42.17
BIC = 50%	1:1 C/I	32.47	27.07
	1.5:1 C/I	32.47	31.07
	2:1 C/I	32.47	37.08
	2.5:1 C/I	32.47	49.11
BIC = 75%	1:1 C/I	35.53	29.47
	1.5:1 C/I	35.53	33.78
	2:1 C/I	35.52	40.25
	2.5:1 C/I	35.52	53.21
BIC = 100%	1:1 C/I	37.56	31.09
	1.5:1 C/I	37.55	35.61
	2:1 C/I	37.55	42.40
	2.5:1 C/I	37.55	56.00

**Fig. 7:**
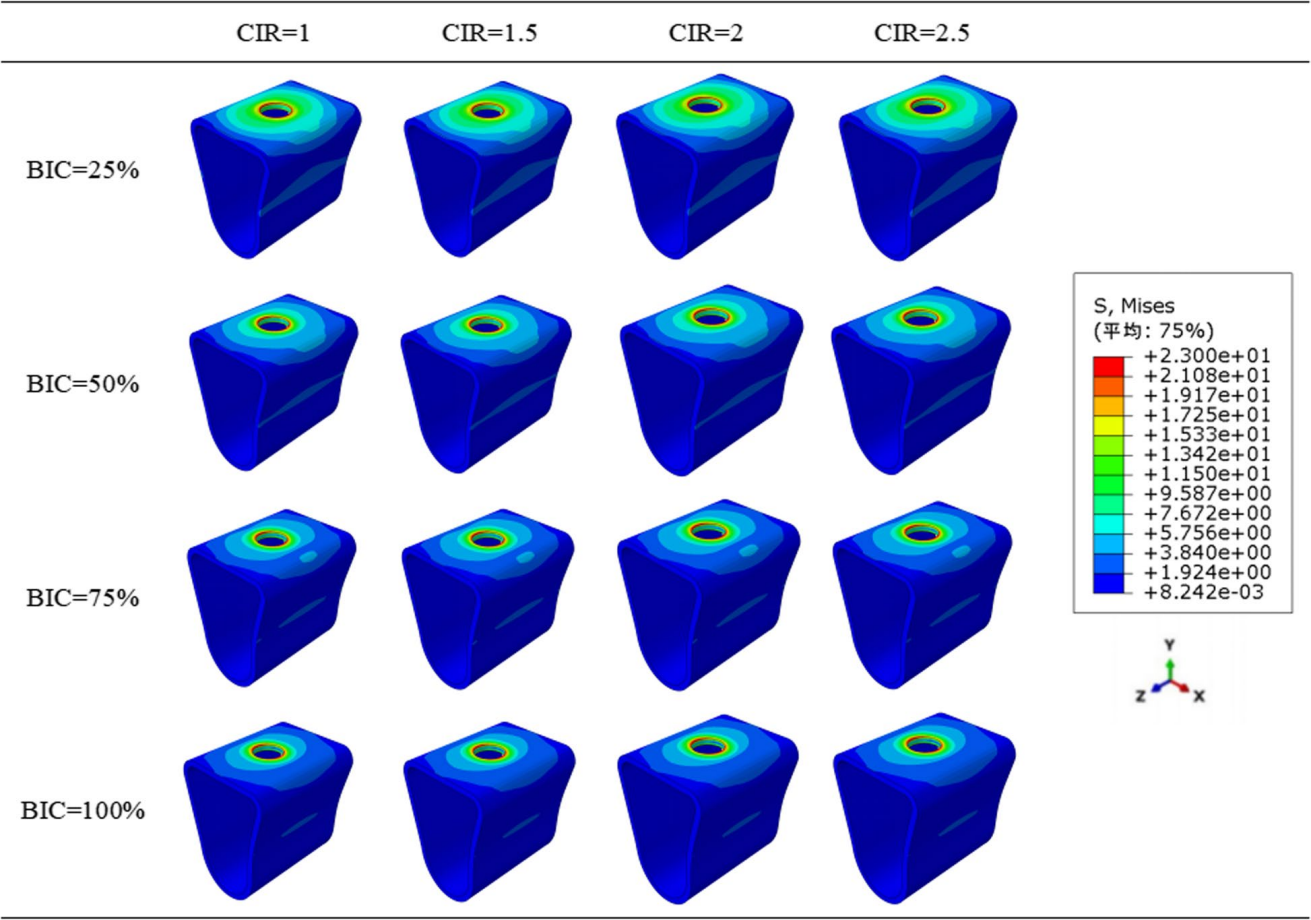
Stress distribution patterns in all the models

**Fig. 8:**
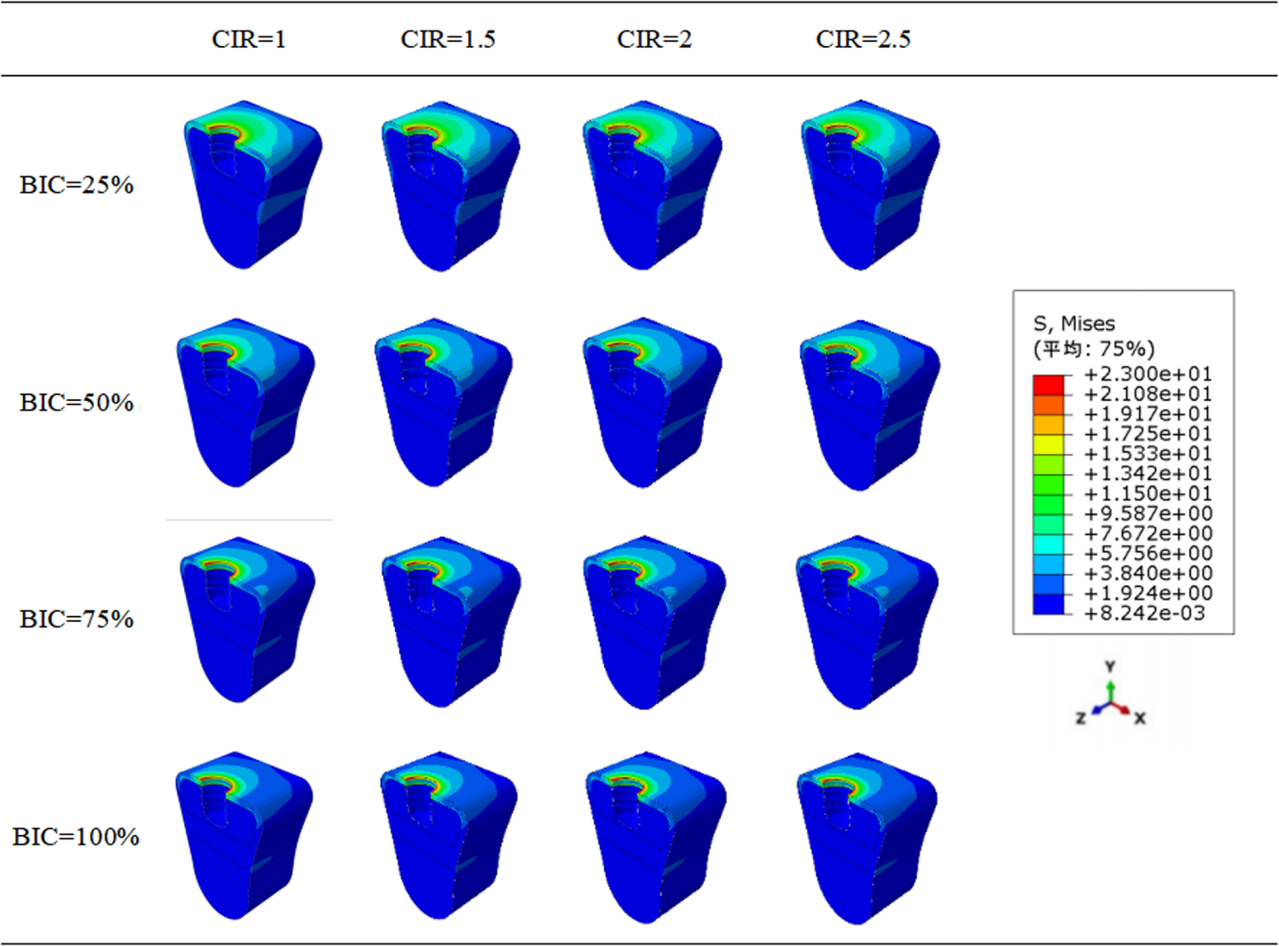
Stress profiles of all the models

According to the stress distribution maps mentioned below (Figs. 7 and 8), the stress was concentrated in the implant neck region in all the models regardless of CIR or osseointegration rates. Furthermore, the stress distribution in the cancellous bone region was uniform.

### Tensile stress, compressive stress, and shear stress

**Fig. 9:**
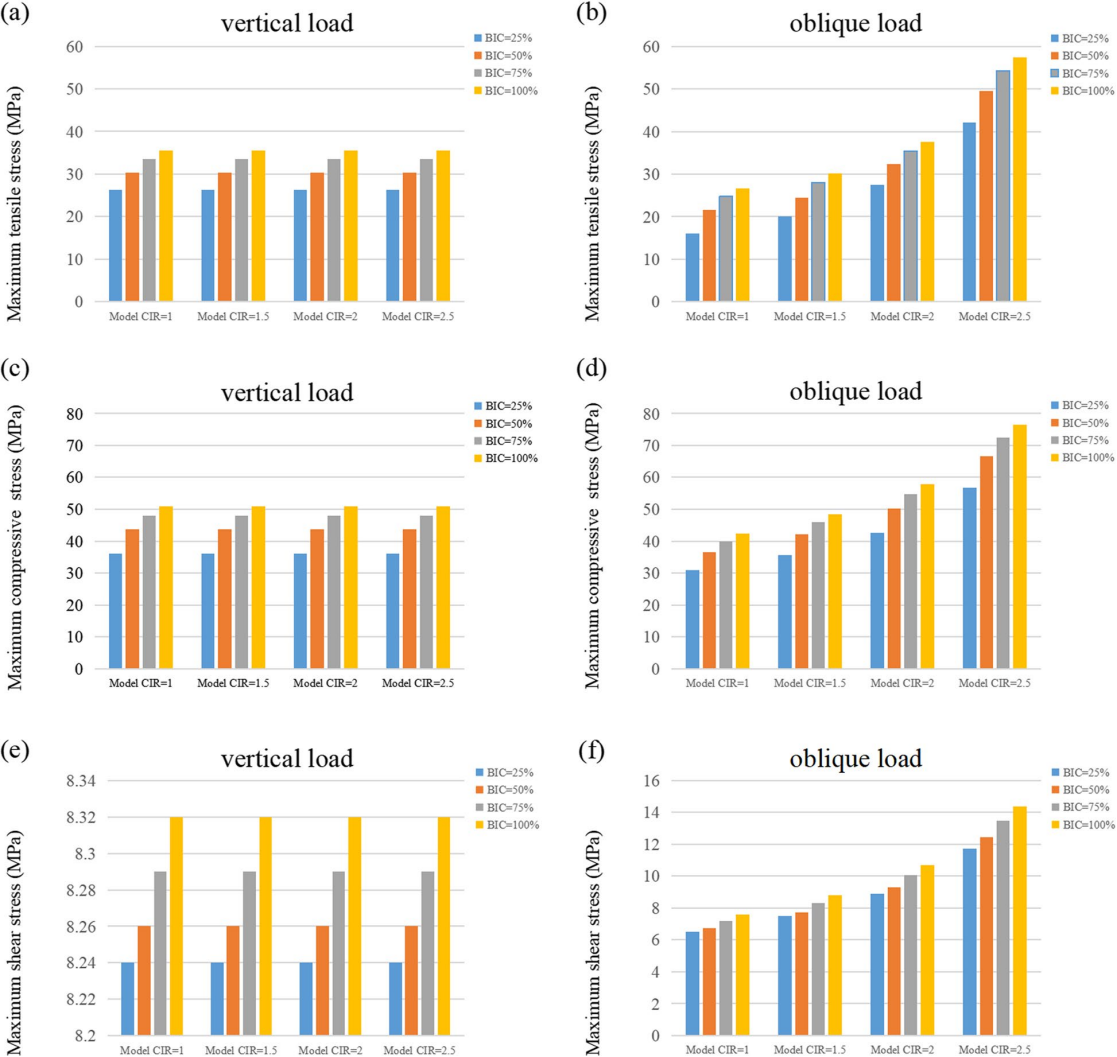
Tensile stress, compressive stress, and shear stress on the cortical bone

As shown in Table 4, the cortical bone’s tensile and compressive stresses were within the required range of tensile and compressive strengths in all the models. Under the vertical loads, the three forces in the models with different CIRs did not show any significant difference. However, under inclined loads, the tensile stress, compressive stress, and shear stress on the cortical bone increased as the osseointegration rate increased, including a 37%-35%, 36–67%, and 16–23% increase in compressive stress, tensile stress, and shear stress, respectively. Moreover, the tensile stress was more susceptible to osseointegration rate than compressive stress. Similarly, tensile stress, compressive stress, and shear stress all increased with the enhancement of CIRs (Fig. 9).

**Table 4:** Tensile stress, compressive stress, and shear stress on the cortical bone

		Maximum tensile stress (MGPa)		Maximum compressive stress (MGPa)		Maximum shear stress (MGPa)	
		Vertical load	Inclined load	Vertical load	Inclined load	Vertical load	Inclined load
BIC = 25%	1:1 C/I	26.31	15.98	36.18	30.87	8.24	6.76
	1.5:1 C/I	26.31	20.07	36.18	35.57	8.24	7.73
	2:1 C/I	26.31	27.43	36.18	42.63	8.24	9.29
	2.5:1 C/I	26.31	42.15	36.18	56.74	8.24	12.45
BIC = 50%	1:1 C/I	30.33	21.61	43.77	36.61	8.26	6.76
	1.5:1 C/I	30.33	24.54	43.77	42.07	8.26	7.73
	2:1 C/I	30.33	32.36	43.77	50.25	8.26	9.29
	2.5:1 C/I	30.33	49.62	43.77	66.64	8.26	12.45
BIC = 75%	1:1 C/I	33.48	24.73	48.01	39.98	8.29	7.17
	1.5:1 C/I	33.49	28.04	48.01	45.89	8.29	8.32
	2:1 C/I	33.49	35.42	48.00	54.77	8.29	10.04
	2.5:1 C/I	33.49	54.24	48.00	72.52	8.29	13.49
BIC = 100%	1:1 C/I	35.60	26.71	50.81	42.28	8.32	7.59
	1.5:1 C/I	35.60	30.25	50.81	48.51	8.32	8.82
	2:1 C/I	35.60	37.59	50.80	57.86	8.32	10.67
	2.5:1 C/I	35.60	57.48	50.80	76.58	8.32	14.38

### Maximum strain

**Fig. 10:**
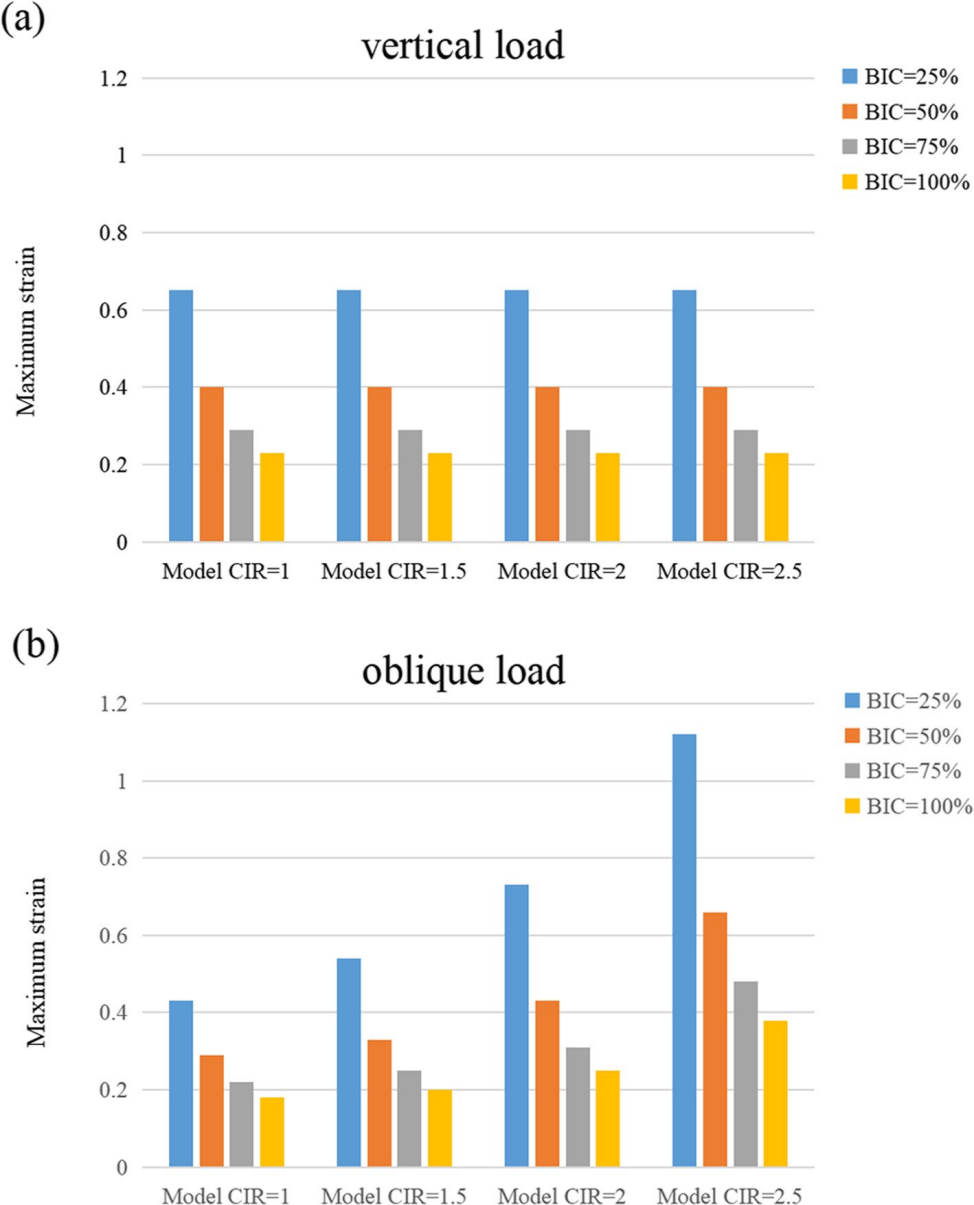
Maximum strain of the cortical bone

According to Fig. 10, the maximum strain of cortical bone did not show any significant difference with different CIRs under vertical loads but displayed significant differences under inclined loads. The maximum strain of the cortical bone under inclined loads increased with the enhancement of CIR. Moreover, at the same CIR, the higher the osseointegration rate, the lesser the maximum strain of the cortical bone (Table 5).

**Table 5:** Maximum strain of the cortical bone

		Maximum strain	
		Vertical load	Inclined load
BIC = 25%	1:1 C/I	0.65	0.43
	1.5:1 C/I	0.65	0.54
	2:1 C/I	0.65	0.73
	2.5:1 C/I	0.65	1.12
BIC = 50%	1:1 C/I	0.4	0.29
	1.5:1 C/I	0.4	0.33
	2:1 C/I	0.4	0.43
	2.5:1 C/I	0.4	0.66
BIC = 75%	1:1 C/I	0.29	0.22
	1.5:1 C/I	0.29	0.25
	2:1 C/I	0.29	0.31
	2.5:1 C/I	0.29	0.48
BIC = 100%	1:1 C/I	0.23	0.18
	1.5:1 C/I	0.23	0.2
	2:1 C/I	0.23	0.25
	2.5:1 C/I	0.23	0.38

### Maximum displacement

**Fig. 11:**
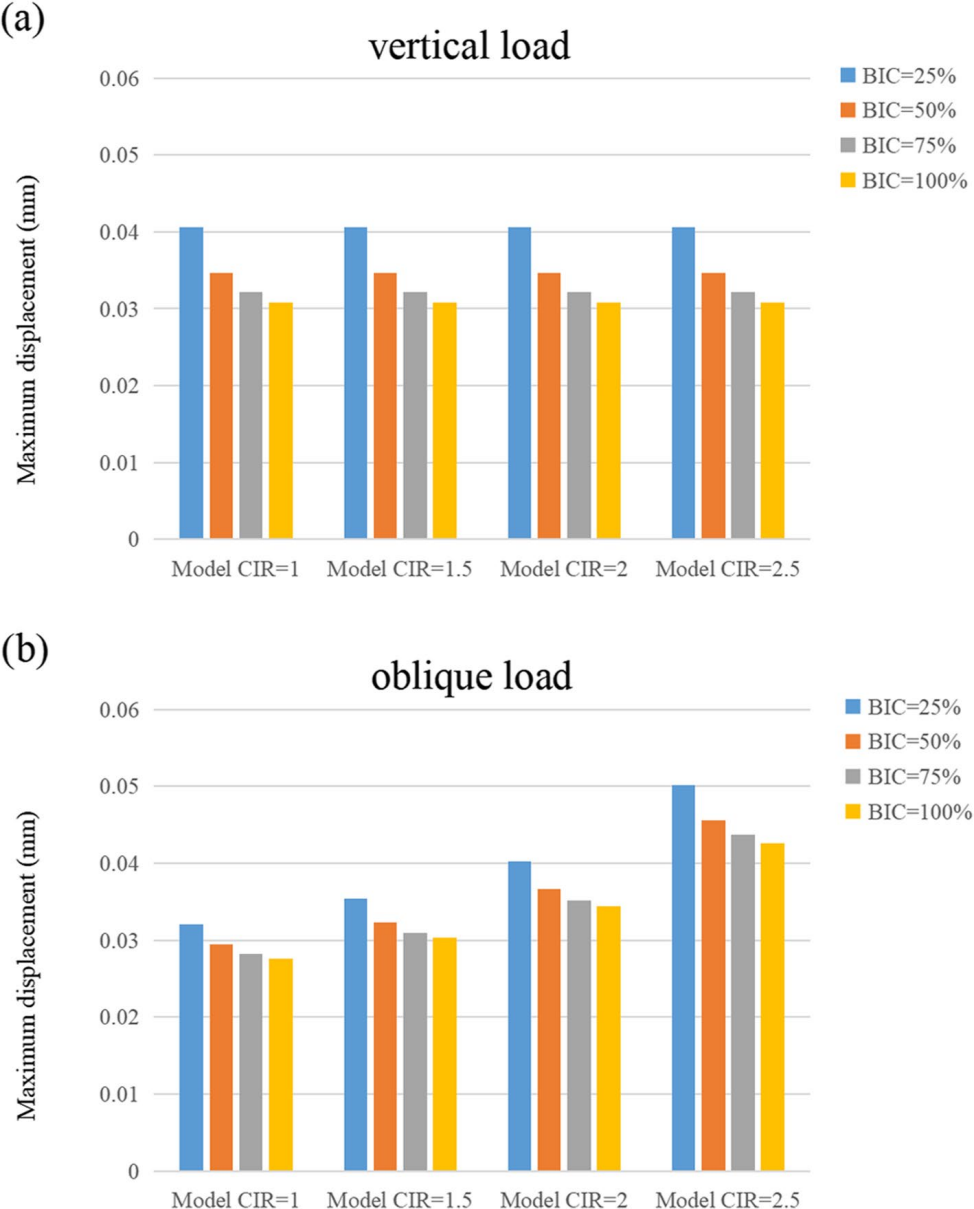
Maximum displacement of the cortical bone

**Fig. 12:**
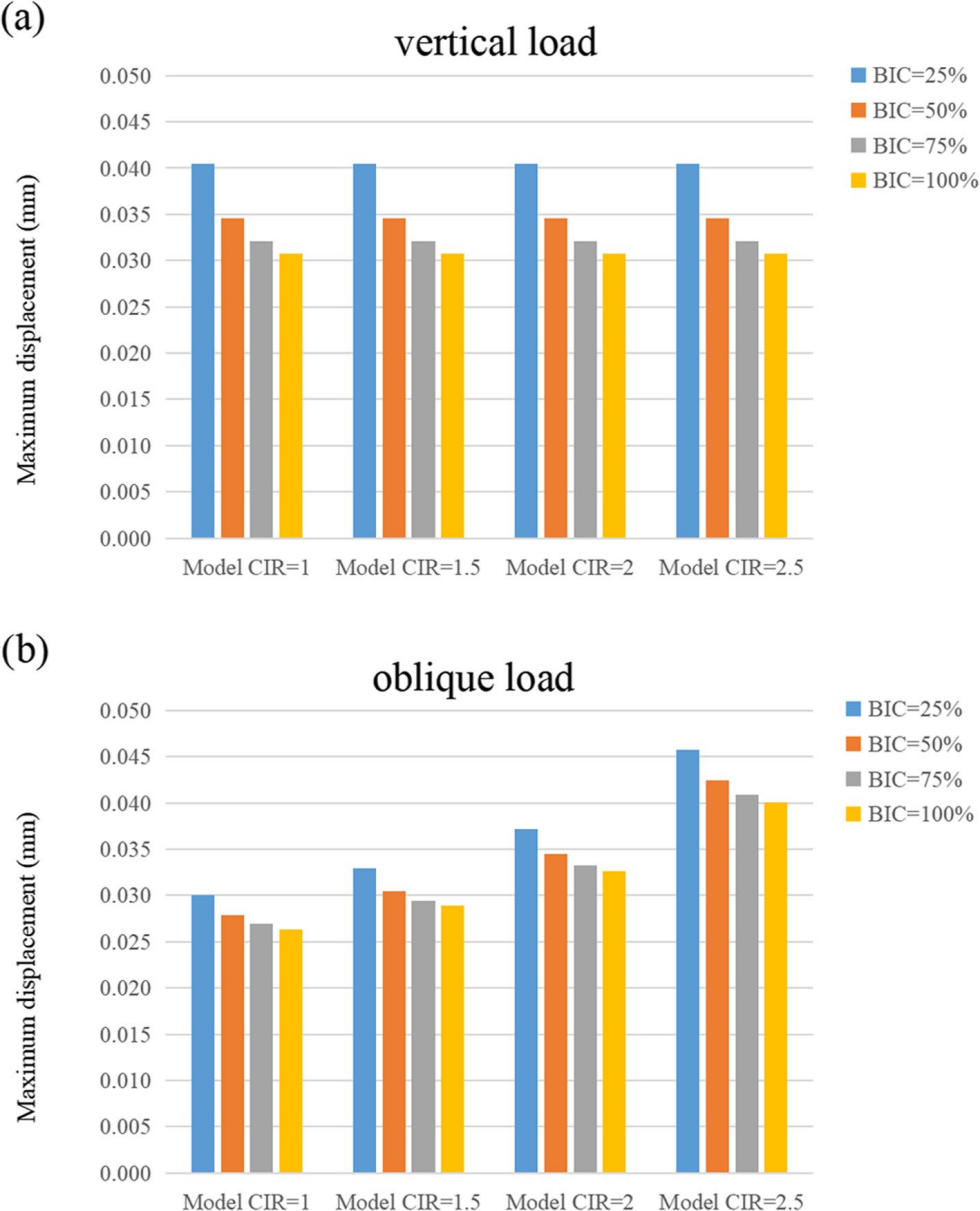
Maximum displacement of the cancellous bone

**Fig. 13:**
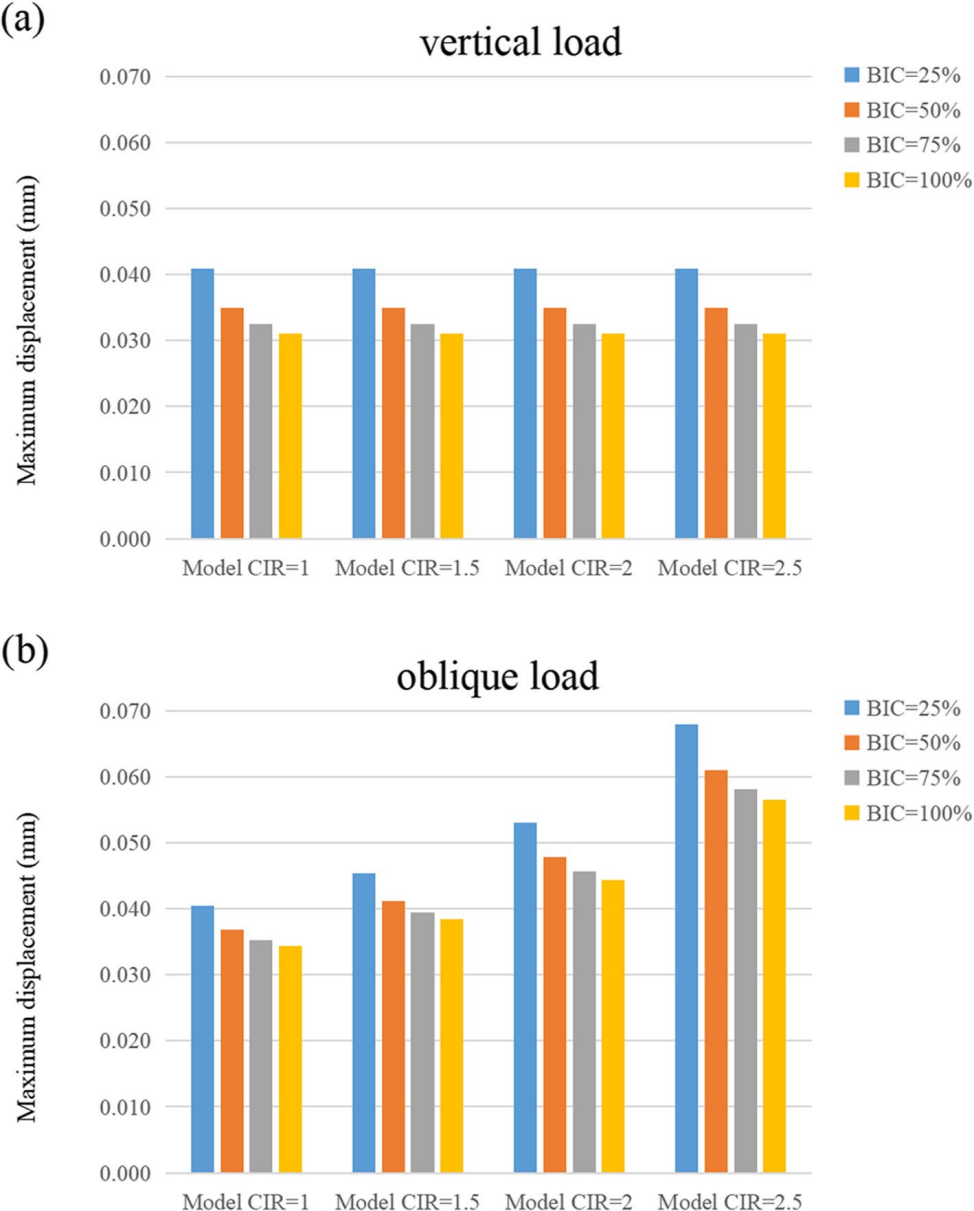
Maximum displacement of the implant

Figures 11, 12, and 13 show the maximum displacement of the cortical and cancellous bones, as well as the implant under vertical and inclined loads, respectively. In all the models, the greatest and the smallest displacement occurred in the implant and the cancellous bone regions, respectively. Although the maximum displacement under vertical loads was similar in magnitude, the maximum displacement increased with enhanced CIR in inclined loads. However, the maximum displacement decreased as the osseointegration rate increased at the same CIR (Table 6).

**Table 6:** Maximum displacement of the cortical bone, cancellous bone, and implant

		Maximum displacement under vertical loads			Maximum displacement under inclined loads		
		Cortical bone	Cancellous bone	Implant	Cortical bone	Cancellous bone	Implant
BIC = 25%	1:1 C/I	0.0406	0.0405	0.0409	0.0321	0.0300	0.0405
	1.5:1 C/I	0.0406	0.0405	0.0409	0.0354	0.0329	0.0454
	2:1 C/I	0.0406	0.0405	0.0409	0.0403	0.0372	0.0530
	2.5:1 C/I	0.0406	0.0405	0.0409	0.0502	0.0458	0.0680
BIC = 50%	1:1 C/I	0.0346	0.0346	0.0349	0.0294	0.0279	0.0368
	1.5:1 C/I	0.0346	0.0346	0.0349	0.0323	0.0305	0.0412
	2:1 C/I	0.0346	0.0346	0.0349	0.0367	0.0345	0.0478
	2.5:1 C/I	0.0346	0.0346	0.0349	0.0456	0.0424	0.0610
BIC = 75%	1:1 C/I	0.0322	0.0321	0.0325	0.0282	0.0269	0.0353
	1.5:1 C/I	0.0322	0.0321	0.0325	0.0310	0.0294	0.0394
	2:1 C/I	0.0322	0.0321	0.0325	0.0352	0.0332	0.0456
	2.5:1 C/I	0.0322	0.0321	0.0325	0.0437	0.0409	0.0581
BIC = 100%	1:1 C/I	0.0308	0.0307	0.0311	0.0276	0.0263	0.0344
	1.5:1 C/I	0.0308	0.0307	0.0311	0.0303	0.0289	0.0384
	2:1 C/I	0.0308	0.0307	0.0311	0.0344	0.0326	0.0444
	2.5:1 C/I	0.0308	0.0307	0.0311	0.0426	0.0401	0.0565

## Discussion

The usage of short implants in posterior regions with insufficient vertical bone height might provide advantages in terms of fewer treatment costs and sessions as well as a lower incidence of complications. However, their application was often accompanied by a higher CIR. Because of the leverage effect, higher CIRs induced enhanced stresses in the implant and the surrounding bones. Consequently, these stresses may cause marginal bone resorption, screw loosening, and implant fracture and severely affect long-term restorative outcomes [32]. A study showed that increased crown height was a more significant factor than decreased implant length in causing implant failure [33]. The short implants frequently utilized in clinical settings are the 6 mm soft tissue level short implants from Straumann. Thus, this study employed the Straumann 6 mm short implant. It increased the crown height while maintaining a constant length to conduct a biomechanical analysis involving distinct models.

Moreover, the implant’s CIR can be divided into clinical and anatomical CIRs. The clinical CIR is the ratio between the distance from the apex to the implant-bone interface and the distance from the implant-bone interface to the bottom of the implant. Additionally, the anatomical CIR incorporates the prosthesis-abutment shoulder as the boundary rather than the implant-bone interface [34]. However, alveolar bone resorption reduced the clinical CIR, whereas anatomical CIR did not change. Therefore, clinical CIR was more reflective of the clinical reality of implants [35]. We used clinical CIR, and the stress distribution at different CIRs was simulated under Class III bone conditions.

Although bone is considered an anisotropic material, its material behavior can further categorize the characteristics as transverse isotropic and longitudinal anisotropic. Hence, we assumed that bone was transverse isotropic to facilitate calculations. The stress values while calculating isotropy were 20%–30% higher than the values obtained in transverse isotropy [36]. When the calculated stress was lower than the bone’s yield strength, it indicated that this value was within the loading range of the bone. Additionally, Kurniawan et al. [16] proposed a theory of different yield strengths at different osseointegration rates and confirmed that the maximum von Mises stress on the cortical bone around the short implant exceeded the yield strength at an osseointegration rate of 25% and a CIR of 2.5. Thus, these results suggested that the implant failure risk was higher when the CIR was ≥ 2.5. Hence, short implants should be carefully selected according to the patient’s conditions and occlusal habits.

Although the stress distribution on the implant and bones was uniform in vertical loading, the maximum von Mises stress distribution pattern did not change significantly with an enhanced CIR. This might have occurred because the direction of force transmission occurred on the implant’s long axis, resulting in a nonsignificant increase in tension [37]. However, the maximum von Mises stresses on the implant and bones were positively correlated with CIRs under inclined loads; this finding was consistent with the results obtained by Ercal et al. [6]. Similarly, Sutpideler et al. [38] also demonstrated that the stress increased as the crown height expanded under inclined loads. Furthermore, our results confirmed that the implants were significantly affected by inclined loads, thereby suggesting that lateral forces should be duly considered in short implant restorations. Moreover, the lateral masticatory forces can be curtailed in patients by reducing their buccolingual diameter and cusp inclination.

Additionally, we proposed that the maximum von Mises stresses on the cortical bone were higher as the CIR increased under non-axial loads. The longer the crown height of the fixed-length implants was, the greater the lever force and the marginal bone loss. Hingsamer et al. [39] revealed that the short implants with a CIR of ≥ 1.7 were prone to marginal bone loss. Additionally, Meijer et al. [15], in a meta-analysis of single-crown restorations supported by short implants, confirmed that no significant differences were observed in retention rate and marginal bone resorption when the single crown CIR was 0.86–2.14. On the contrary, Garaicoa-Pazmiño et al. [12], in a systematic review, concluded that when the CIR was 0.6–2.36, the greater the CIR, the lower the bone resorption. They suggested a high CIR promoting bone remodeling activity might be preventing marginal bone resorption [40, 41]. Another study by Takahashi et al. [31] concluded that augmentation surgery was not required in cases where the crown height was < 15 mm; the reduced crown height helped in proper stress distribution in the bone around short implants. However, the aforementioned literature did not consider the crown height, which might be one of the reasons for varying conclusions. Our results suggested that the short implants were successful at higher osseointegration rates with a CIR of 2.5. Therefore, short implants with high CIR can achieve higher success rates in controlled occlusal and parafunctional habits, thereby allowing the establishment of a reasonable crown height.

In general, occlusal masticatory forces generate three different forces at the implant-bone interface; tensile stress, compressive stress, and shear stress [21]. Compressive stress can enhance bone strength, while tensile stress can pull apart or stretch the material. Therefore, the ideal biomechanical environment requires a balance between tensile stress and compressive stress. Since shear stress promotes slippage at the bone-implant interface, it is the least unfavorable force for implant stability. Our results suggested that although both tensile and compressive stress increased with enhanced osseointegration rate, tensile stress was more easily affected by the osseointegration rate. This may be due to the synergism between osseointegration and compressive stresses. Furthermore, the magnitude of shear stress increased as the CIR increased, indicating that the greater the CIR, the enhanced the likelihood of implant slippage. Therefore, implant design factors, such as thread shape, should be considered to minimize the shear stress when choosing shorter implants with large CIRs.

Our results showed that the maximum von Mises stress and strain on the cortical bone increased and decreased as the osseointegration rate increased at the same CIR. These findings demonstrated that deformation was required for bones with a low osseointegration rate to compensate for such loads. Additionally, the stress–strain relationship was a form of energy (the area under the stress–strain curve equaled the strain energy), which suggested that osteocytes with lower osseointegration rates needed Simultaneously, Kurniawan et al. [16] proposed that different osseointegration rates corresponded to different yield strengths, and higher stresses were tolerable for bones with higher osseointegration rates as their stress values were lower relative to their yield strengths. Additionally, the maximum displacement negatively correlated with the osseointegration rate under inclined loads.

The maximum von Mises stress distribution patterns in our study showed that the maximum stress was concentrated in the implant neck region in all models and was consistent with previous studies. In natural teeth, the presence of periodontal ligament provides certain mobility, thereby avoiding the stress concentration on the cortical bone. However, as osseointegrated implants are attached to the bone through surface micropores with a rigid interface, the external loads are directly transmitted to the bone without buffering; therefore, stress is often concentrated around the implant neck area.

There were some limitations in our study. Although this 3D FEA was a simulation study and the numerical model was appropriately simplified, the complex oral environment cannot be reproduced distinctly. Biological systems exhibit the capacity to adapt in response to external stimuli. Thus, when employing the finite element method within the context of biological medicine, some disparities from actual situations may arise [18]. Additionally, despite acknowledging the existence of transverse isotropic bone properties, bone is still represented as a linear elastic continuum. It is noteworthy that the mandible notably displays orthogonal anisotropy and viscoelastic behavior. Furthermore, the interplay between bone and implant changes over time. These variables collectively influence the experimental outcomes to a certain extent. This study is unable to entirely replicate real clinical scenarios and can solely offer theoretical data to substantiate biomechanical aspects. The utilization of CBCT data can facilitate the construction of precise models, enhancing the alignment of the model with clinical practice [42]. Concurrently, we will pursue ongoing clinical investigations to validate these findings. Additionally, there is a lack of studies on stress distribution with crown heights > 15 mm, which should be further explored.

## Conclusion

Our study yielded the following conclusions:

1. When the osseointegration rate is the same, there is no significant difference in the stress values among various crown-to-implant ratios under vertical loading. However, under oblique loading conditions, the crown-to-implant ratio demonstrates a positive correlation with stress values. When opting for short implants, efforts should be made to minimize lateral forces.
2. When the crown-to-implant ratio reaches 2.5 or higher, it is imperative to thoroughly assess the patient's individual circumstances and exercise caution when contemplating the use of short implants.

## Data Availability

All data generated and analyzed during this study are included in this article.
